# Reversible Thyroid Dysfunction Associated With Quetiapine Use in an Adolescent Patient: A Case Report

**DOI:** 10.7759/cureus.86845

**Published:** 2025-06-27

**Authors:** Pushyami Satya Bandi, Khalil-Uz-Zaman Qadri, Adiraj Singh, Deiaa Elhag

**Affiliations:** 1 Internal Medicine/Pediatrics, Hurley Medical Center/Michigan State University (MSU), Flint, USA; 2 Psychiatry and Behavioral Sciences, Hurley Medical Center/Michigan State University (MSU), Flint, USA

**Keywords:** adolescent female, quetiapine, second-generation antipsychotics, sub-clinical hypothyroidism, thyroid dysfunction

## Abstract

Hypothyroidism frequently manifests with neuropsychiatric symptoms that may overlap with primary psychiatric disorders. Quetiapine, an atypical antipsychotic commonly used in mood and psychotic disorders, is generally well tolerated but has been associated with metabolic disturbances and, less commonly, thyroid dysfunction such as subclinical hypothyroidism. We report the case of an adolescent female treated with quetiapine for mood instability and suicidality, who developed elevated thyroid-stimulating hormone (TSH) levels and dyslipidemia despite remaining clinically euthyroid. Dose reduction and eventual discontinuation of quetiapine led to normalization of thyroid function and lipid profile, while psychiatric stability was maintained. Possible mechanisms for quetiapine-induced thyroid dysfunction include dopamine receptor antagonism affecting TSH regulation, direct thyroid effects, and metabolic changes related to weight gain. This case highlights the necessity for routine monitoring of thyroid and metabolic parameters during quetiapine therapy to ensure optimal management of psychiatric symptoms while minimizing endocrine complications.

## Introduction

Hypothyroidism is a condition characterized by insufficient production of thyroid hormones, leading to symptoms such as fatigue, weight gain, depression, and cognitive impairment [1,2]. Subclinical hypothyroidism refers to an early stage of thyroid dysfunction where elevated thyroid-stimulating hormone (TSH) levels are present, but thyroid hormone levels remain normal, often without noticeable symptoms [3,4].

Hypothyroidism, a common endocrine condition affecting roughly 3.6% of the population, presents a range of clinical symptoms, including significant neuropsychiatric manifestations such as sadness, anxiety, mania, and cognitive impairment [1,2]. Fatigue, weakness, and anhedonia or apathy - common clinical features of hypothyroidism - can be misattributed to depression or the negative symptoms of schizophrenia [3-5]. Consequently, hypothyroidism may be overlooked during the acute management of schizophrenia [3]. Several pharmacologic agents have been associated with drug-induced hypothyroidism. Quetiapine, an atypical antipsychotic, has been reported to reduce thyroid hormone levels in certain individuals [6]. Quetiapine, a second-generation antipsychotic, is extensively used to treat schizophrenia, bipolar disorder, and depression. It is popular because of its tolerance and low likelihood of extrapyramidal effects. Due to its relatively favorable tolerability profile, it is frequently prescribed for a variety of psychiatric conditions, including schizophrenia, bipolar disorder, and major depressive disorder. However, despite its broad utility, emerging evidence suggests that quetiapine may influence thyroid function, potentially leading to clinically significant hypothyroidism in susceptible individuals. In this report, we describe the case of a patient who developed hypothyroidism subsequent to the introduction of quetiapine into her treatment regimen, highlighting the importance of monitoring thyroid function during antipsychotic therapy [7].

## Case presentation

An adolescent female with no prior psychiatric history presented to the emergency department following a suicide attempt. Upon admission to the inpatient psychiatric unit, she was alert, hemodynamically stable, and oriented. Mental status examination revealed active suicidal ideation, disrupted sleep, and a persistently depressed affect. She denied any perceptual disturbances and did not express homicidal ideation. Initial laboratory investigations, including thyroid function and lipid profile, were within normal limits.

The patient was initiated on citalopram 10 mg daily and quetiapine 50 mg at bedtime. Quetiapine was gradually titrated to 300 mg/day during her inpatient stay. Due to limited improvement in depressive symptoms, citalopram was discontinued, and bupropion 150 mg daily was introduced. Based on clinical notes, the combination of bupropion and quetiapine was linked to a significant improvement in symptoms, such as the cessation of suicidal thoughts, better sleep, and functioning. The patient was discharged on 300 mg of quetiapine per day and trazodone for sleep.

In outpatient follow-up, quetiapine was increased to 400 mg/day due to ongoing mood instability. Bupropion and trazodone were subsequently discontinued. She maintained clinical stability on the increased dose of quetiapine, demonstrating a favorable response to monotherapy.

After several months on this regimen, routine laboratory testing revealed abnormalities in thyroid function and lipid metabolism. The patient’s thyroid panel showed a TSH of 9.8 μIU/mL with a free T4 of 0.6 ng/dL, consistent with biochemical hypothyroidism. The lipid profile demonstrated a total cholesterol of 242 mg/dL, low-density lipoprotein (LDL) of 160 mg/dL, and triglycerides of 210 mg/dL. Despite these findings, the patient remained asymptomatic for hypothyroidism, reporting no typical symptoms such as weight gain, fatigue, cold intolerance, or constipation. Given the clinical picture, drug-induced subclinical hypothyroidism was suspected. Quetiapine was continued due to sustained psychiatric stability; however, the dose was reduced to 300 mg/day in consideration of emerging evidence suggesting dose-dependent effects on thyroid function, with the aim of mitigating potential thyroid and metabolic disturbances while maintaining mental health gains; however, on repeat testing thyroid function abnormalities persisted. Thyroid peroxidase (TPO) antibody levels were subsequently tested and found to be negative, reducing the likelihood of autoimmune thyroiditis. As a result, quetiapine was gradually tapered and fully discontinued over the course of one month. The patient continued psychotherapy and remained psychiatrically stable throughout this process.

At follow-up several months after discontinuation, thyroid function and lipid levels had normalized, supporting a diagnosis of reversible, quetiapine-induced subclinical hypothyroidism. Lipid profile testing was unavailable at the 6-month follow-up due to the patient’s loss to follow-up; however, thyroid function tests were obtained and confirmed through medical record review. A summary of the patient’s thyroid function tests and lipid measurements throughout the treatment course, are presented in Table 1, highlighting the temporal relationship.

**Table 1:** Laboratory findings Including thyroid function tests and lipid panel values over the course of treatment. Reference ranges for each parameter are provided. Arrows (↑) indicate values above the normal reference range. TSH: thyroid-stimulating hormone; LDL: low-density lipoprotein; HDL: high-density lipoprotein

Time Point (Quetiapine Dose)	TSH (mIU/L) (N: 0.4–4.0)	Free T4 (ng/dL) (N: 0.8–1.8)	Total Chol (mg/dL) (N: <200)	HDL (mg/dL) (N: >40)	LDL (mg/dL) (N: <100)	Triglycerides (mg/dL) (N: <150)
Baseline (0 mg/day)	1.14	Normal	152	44	94	69
3 Months Post-Initiation (400 mg/day)	7.4 ↑	1.2	218 ↑	52	149 ↑	87
After Dose Reduction (300 mg/day)	8.26 ↑	1.3	253 ↑	52	183 ↑	91
6 Months Post-Quetiapine (0 mg/day)	2.3	Normal	Not assessed	Not assessed	Not assessed	Not assessed

## Discussion

This case illustrates the challenges of managing mood instability and suicidality in adolescents, especially in the setting of significant trauma and a notable family history of psychiatric disorders. Quetiapine was effective in stabilizing mood, but its use was complicated by metabolic and endocrine side effects, including increased cholesterol, and LDL, and thyroid dysfunction. These adverse effects necessitated close monitoring and dosage modifications to optimize symptom control while minimizing potential long-term health consequences.

Quetiapine is a second-generation (atypical) antipsychotic agent that exhibits a greater affinity for serotonin 5-HT₂A receptors than for dopamine D₂ receptors, a characteristic that contributes to its antipsychotic efficacy while minimizing the risk of extrapyramidal side effects and is frequently used for bipolar disorder, schizophrenia, and major depressive disorder. Despite its generally favorable tolerability, it has been linked to metabolic side effects such as weight gain, dyslipidemia, and glucose metabolism disturbances [8-10]. In this case, the patient did not report any significant weight gain or changes in glucose parameters throughout the treatment course, which contrasts with the typical metabolic risks observed in the literature. This absence of such side effects highlights the variability of quetiapine’s metabolic impact and emphasizes the importance of individualized monitoring. Less frequently, quetiapine has been implicated in thyroid dysfunction, particularly subclinical hypothyroidism [3,11].

Subclinical hypothyroidism is defined by elevated TSH levels in the presence of normal thyroid hormone concentrations (free T4 and free T3). Although often asymptomatic, it can progress to overt hypothyroidism, particularly in individuals with predisposing factors like autoimmune thyroid disease or a family history of thyroid conditions. Because adolescence is an important time for growth and brain development, it’s important to regularly check thyroid function to support healthy development and prevent any problems.

**Figure 1:**
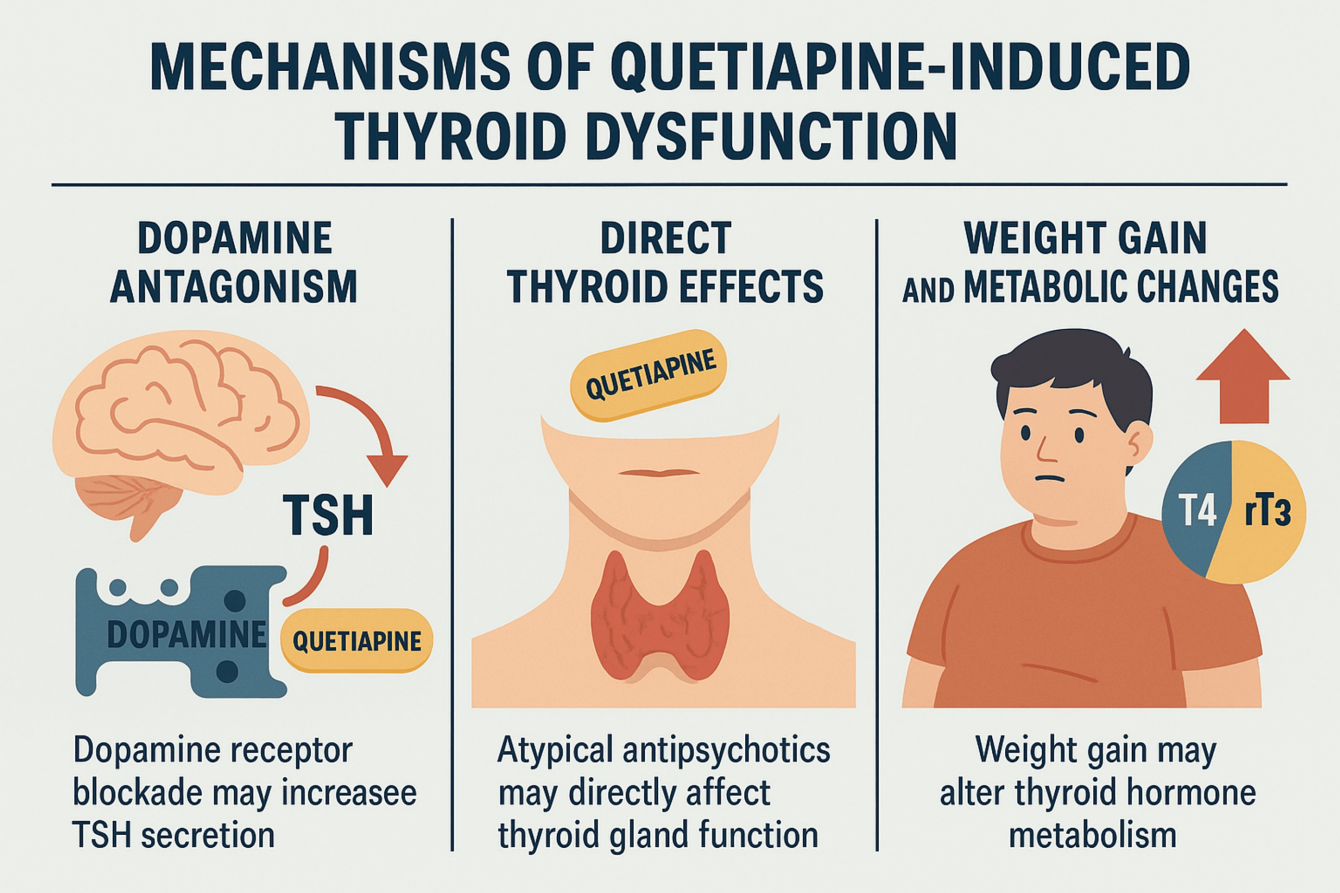
Proposed mechanisms of quetiapine-induced thyroid dysfunction. Schematic representation of potential pathways contributing to quetiapine-associated thyroid dysfunction, including altered hypothalamic-pituitary-thyroid (HPT) axis regulation, possible direct inhibition of thyroid hormone synthesis, and secondary metabolic effects such as weight gain.

The mechanisms by which quetiapine may induce thyroid dysfunction are not fully understood, but several plausible pathways have been proposed. One theory involves its dopamine receptor antagonism, which may disinhibit TSH secretion by removing dopamine’s inhibitory effect on the hypothalamic-pituitary-thyroid axis [3]. Another possibility is a direct effect on thyroid hormone synthesis or secretion, as some atypical antipsychotics have been shown to interfere with thyroid gland function [11]. Additionally, quetiapine-associated weight gain and metabolic changes may alter peripheral hormone metabolism-for instance, by increasing the conversion of thyroxine (T4) to biologically inactive reverse triiodothyronine (rT3)-which can contribute to a functional hypothyroid state despite normal hormone production [9,10,12]. The potential mechanisms by which quetiapine may induce thyroid dysfunction are illustrated in Figure 1.

Given the patient’s elevated TSH alongside normal free T4 and dyslipidemia, quetiapine dosage was gradually reduced to address these side effects while maintaining mood stability. Thyroid dysfunction with quetiapine has been reported to be dose-dependent, with higher doses posing greater risk [3]. Several reports have documented reversal of thyroid abnormalities following dose reduction or discontinuation, supporting a potentially reversible mechanism [6,7,11]. In this case, tapering was preferred over switching antipsychotics due to psychiatric stability. However, in our case, thyroid abnormalities persisted despite dose reduction and only normalized after gradual tapering and complete discontinuation, suggesting that partial dose decrease may not always be sufficient to reverse dysfunction. As quetiapine was tapered, dopaminergic activity may have been restored, potentially normalizing TSH levels indicating the dysfunction was medication-related.

This case emphasizes the necessity for a personalized and adaptable approach to psychiatric medication management in young patients, prioritizing both mental health and physical well-being.

## Conclusions

Quetiapine use in adolescents may contribute to reversible subclinical hypothyroidism and metabolic disturbances, even in the absence of symptoms. This case highlights the need for regular laboratory monitoring during treatment and supports a careful balance between psychiatric benefit and physiological impact. However, the limitations of this single case report must be acknowledged, particularly its lack of generalizability. Larger studies are needed to further validate these findings and explore the underlying mechanisms of quetiapine-associated endocrine and metabolic disturbances. Given these risks, routine thyroid function testing at treatment initiation and during follow-up is recommended to enable early detection and management of thyroid abnormalities. This proactive approach is especially important during adolescence, a critical period for growth and development.
